# Psychotherapeutic Playback Theatre, Well-Being, and Psychological Distress: An Impact Study

**DOI:** 10.3390/ijerph21101288

**Published:** 2024-09-26

**Authors:** António-José Gonzalez, Margarida Pedroso de Lima, Luís Preto, Paulo Martins

**Affiliations:** 1 AppsyCI, Ispa, University Institute, Rua Jardim do Tabaco, 34, 1140-041 Lisboa, Portugal; lpreto@ispa.pt; 2 CINEICC, Faculdade de Psicologia e de Ciências da Educação, Universidade de Coimbra, Rua do Colégio Novo, 3000-115 Coimbra, Portugal; mplima@fpce.uc.pt; 3 Laboratory of Sport Psychology, CIPER, Faculdade de Motricidade Humana, Universidade de Lisboa, Estrada da Costa, 1499-688 Cruz-Quebrada, Portugal; pmartins@fmh.ulisboa.pt

**Keywords:** Psychotherapeutic Playback Theatre, creative arts therapies, group therapy, well-being, psychological distress, therapeutic impact

## Abstract

Psychotherapeutic Playback Theatre (PPT) is a new psychotherapeutic format inspired by Playback Theatre and several therapies. The research presented here aims to study the impact of PPT on the Well-being and Psychological Distress of participants in this expressive-based group psychotherapy. To achieve this, after training 30 psychotherapists and creating an implementation handbook for their use, the research team assisted 20 of them (individually or in groups of two or three) in implementing therapeutic groups that offered 12 weekly sessions, each lasting two to three hours. Nine groups were formed, including a total of 50 participants, who were assessed before and after the PPT program using questionnaires evaluating Psychological Distress and Well-being. The latter consisted of six subscales: Autonomy, Environmental Mastery, Personal Growth, Positive Relations with Others, Purpose in Life, and Self-Acceptance. A control group of 50 participants was assessed using the same variables. No significant differences were found between pre- and post-tests in the control group. However, significant differences were observed in the PPT group, with Psychological Distress decreasing and Well-being improving, both on the total scale and across all subscales except for Self-Acceptance. This set of results allows us to establish connections between participation in the PPT sessions and the positive psycho-emotional effects on participants.

## 1. Introduction

Art can be therapeutic. In the title of a well-known publication about the uses of art, Botton and Armstrong [1] miss one letter to state something that academics [2] and even institutions such as the World Health Organization (WHO) [3] have been showing: that art is (Botton and Armstrong’s book title uses the article “as” instead) therapy. In this particularly aesthetic piece of literary and visual art, the authors list what they call the seven functions of art: remembering, hope, sorrow, rebalancing, self-understanding, growth, and appreciation [1]. Those of us who work in the field of psychotherapy can most probably link each of these functions to psychotherapeutic outcomes or connected themes.

**Figure 1:**
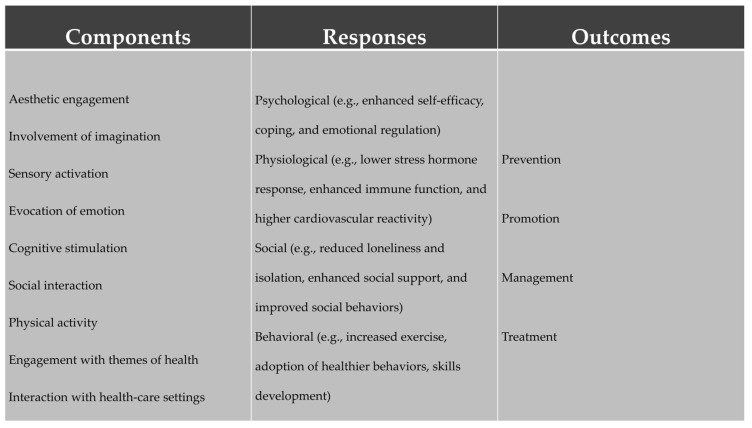
A model of the connections between arts and health [3].

The benefits of the arts as mediators of change in the well-being of individuals are well established. Both in the special issue dedicated to the psychological and physiological benefits of the arts [2] and in the scoping review published by WHO [3], the impact of the use of arts on the well-being of both individuals and communities is well documented. Figure 1, taken from Fancourt and Finn’s work [3], shows in a very clear way the different components involved when arts-based activities are put into action by individuals, groups, and communities.

The use of the arts as therapeutic mediators has been increasingly studied over the last few decades [4,5]. Be it with the use of expressive mediators such as music [6,7], plastic/visual arts [8,9], theatre [10,11,12], dance and movement [13,14], or other forms, and working with patients/participants such as oncological patients [15,16,17,18], people who have schizophrenia [8] or dementia [6,19], palliative care inpatients [15], people with non-psychotic mental health disorders [20], or with other clinical or non-clinical cases [21], inmates [22,23], students (graduate and undergraduate) [21,24], the effects of the use of these mediators in diminishing anxiety [8,9,11,21,24], and depression symptoms [7,8,9,11,13,14,21,24,25], trauma symptoms [24,26], positive symptoms [8,9,11,19,27], among many others, are well documented.

Furthermore, patients usually have a positive opinion of their art-based therapy processes [16,27,28]. However, some show some concerns related to aspects such as not being helpful [16], increasing anxiety and pain [15], and activating emotions that were not dealt with subsequently [17]. It is important to notice that several studies could not find differences either between groups or in the same group before and after the application of art-based treatment [19,29,30]. Some authors pinpoint the importance of studying and reflecting on the harm that can be caused by this (and any other) form of psychotherapy [8,31].

### 1.1. Theatre as Therapy

The several components connected to the use of arts in this context, involving multiple kinds of individual responses, result in outcomes that include prevention, promotion, management, and treatment. In the case of theatre-based therapies or applications—some authors use the terms “applied theatre” or “applied drama”, with these including Oppressed Theatre, Educational Theatre, and other uses of theatre/drama to enhance well-being (see the meta-analysis by Lewandowska and Węziak-Białowolska [32])—such as psychodrama, drama therapy, therapeutic theatre, or psychotherapeutic playback theatre. We would stress the presence of some therapeutic elements, such as aesthetic engagement (and the properties of the aesthetic space, as conceptualized by Boal [33]); the use of imagination and the cognitive plasticity it brings; the use of the body in its several potentials, including accessing knowledge, intuition, flexibility, expressivity, among others, and, of course, the social encounters and interactions these dramatic spaces allow for. Altogether, the multiple aspects brought to life in the dramatic spaces explored in therapeutic contexts are strong tools for personal change.

Be it with the use of theatre with students or trainees [34,35,36], and specifically with students of health sciences (see the systematic review by Bas-Sarmiento et al. [37]), the effects of dramatherapy with children, adolescents, or adults [38,39,40,41], or in the case of psychodrama [11,42,43,44,45], the literature has consistently shown the efficacy of this kind of intervention with different populations and in various health indicators. In the next section, we shall introduce the case of a particular and new form of theatre-based therapy, psychotherapeutic playback theatre (PPT).

### 1.2. From Playback Theatre to Psychotherapeutic Playback Theatre

The recent psychotherapy format that inspired our work is based on Playback Theatre (PT), created by Jo Salas and Jonathan Fox [46,47,48,49], in the 1970s, and several therapeutic approaches, like group analysis, dramatherapy, narrative therapy, and psychodrama [46,50]. Playback Theatre, the main inspiration for PPT, is an improvised theatre format that draws from the narratives of the audience members, which inspires playbackers and musicians to create an aesthetic experience that, at the same time, respects and reframes the original stories [46,50,51].

Although, when one thinks of PT, the most probable image that will come to mind is that of a theatrical performance, it is in the intimacy of the troupe and its typically weekly rehearsals that the (therapeutic) benefits of this now 50-year-old practice are best felt. From our own practice as playbackers and the exchanges with our playback community colleagues, we realize that the practice of meeting once a week, in a closed circle, sharing our true and personal stories—whether from the same day they are told or from our childhood, our dreams, our fantasies, or any other areas of our lives—is frequently associated with experiences of relief, creative reframing, feeling understood, hopefulness, empathy, and well-being, among many other significant experiences. Furthermore, this applies not only to the tellers but also to the performers and the audience. It was actually this kind of experience that led the creators of PPT (Kowalsky, Keisari, and Raz) to step forward with a new therapy [18].

The therapeutic effects of PT have, therefore, been a topic of discussion among the community of playbackers since its very beginning [52]. A recent literature review that focused on the publications dealing with the application of PT found 79 articles published in the last three decades, 18 of which were evaluation-centered and in English [5]. In doing so, most of the above-mentioned articles assessed variables directly connected to therapeutic effects, such as empathy, anxiety, cognitive functioning, depression, PTSD, and spontaneity, among others.

Psychotherapeutic playback theatre was, therefore, created in order to enhance some of these therapeutic effects. The main differences between PPT and PT, in addition to the obvious difference in intention, are the absence of an external audience (which is also common in PT rehearsals), the presence of one or more psychotherapists with specific training, and the multiple roles participants take during a session which vary between telling a story, performing as playbackers (or musicians) or witnessing the performance of others [18]. Several articles have been recently published concerning the effects of PPT on institutionalized elders, showing benefits in general mental health, creativity, and life meaning [53,54,55]. To our knowledge, there are no published studies concerning the use of PPT with other populations.

From the other psychotherapies that inspired it, PPT either took or adapted some of their respective main mechanisms of change. The transformative power of stories and narratives has a long-term presence in both the popular (we always remember the life-saving—for Scheherazade—and therapeutic—for King Shahryar—effects of the one-story-a-night practice that constitutes the core of *The Arabian Nights*) and scientific cultures [56,57,58,59,60]. Authors like Erik Erikson [61], Michael White and David Epston [56], and Dan McAdams [59], among many others, have shown how human change processes are interwoven with the changes in the narratives associated with life processes.

Moreno, the creator of psychodrama and group psychotherapy [62] and another inspiration for PPT, brings action (drama) and spontaneity [63] to the very core of psychotherapeutic change. In addition to sharing, by telling or narrating our personal stories, acting through dramatization with other group members opens new perspectives for people looking for personal change [64,65]. We owe Moreno the idea that spontaneity itself is a main mechanism of therapeutic change, just as we owe him the strengthening of the connection between theatre and psychotherapy.

Drama therapy also invests in the idea of using drama to deal with life situations in therapeutic settings. Among its principles, the use of dramatic distance, projective identification, empathy, and embodiment, among others [66,67], is present in PPT sessions. 

Last but surely not least, group analysis is at the very center of the creation of PPT. Both the titles of the seminal book [68] and scientific paper [68] from the creators of PPT include a reference to Foulkes’s concept of *Hall of Mirrors* [69,70,71]. The main idea of this concept is that others can reflect us in a myriad of ways, from empathetic to confrontational, and the group therapists have to frame them, for they can have both benign and malignant effects. In PPT, seeing others reflect parts of ourselves and parts of our narratives can be a strong experience that can help participants reframe their stories, bring awareness to parts of themselves, or externalize and concretize internal parts of their experiences (see the concept of concretization and its multiple therapeutic uses in the work of Kushnir and Orkiby [72]).

### 1.3. The Progression of a Psychotherapeutic Playback Theatre Session

A PPT session, like some of the therapies that influenced it, should start with a group ritual, generally in a circle, to mark the passage from the social context to the group one. The warm-up phase should help connect the group members, enhance body and mind availability to both listen and act and, sometimes, connect to previous sessions or themes considered important to the group. The therapeutic team can use several group dynamics to achieve these goals. 

Stories are at the core of PPT, and sooner rather than later, time and space should be given to personal narratives. Now that the participants are warmed up, someone—ideally spontaneously—will start sharing a story with the group, whether it is from that moment (about re-encountering the group, for example), that day, that week, or any other source of memory. Stories should be personal and true. 

In this new phase of the session, the therapist leading the session should guide the teller in preparing what is called a “short form”. In this first contact with dramatization, several participants are invited to, after thoroughly listening to the teller, re-enact the story in a very simple way. Examples of short forms include variants of the Playback Theatre’s most typical fluid sculpture, machine, or snapshot forms (for a more detailed description of these and other forms, the toolkit by Elinger and Elinger [51] is an excellent source). The idea is that either one by one or all together, the participants who listened to the narrative will bring a part of that narrative to the stage in the form of a (repetitive) gesture, sound, word, or any other form of representation, creating a very short and naïve scene that honors the teller’s sharing [51,73]. We are now entering what, in psychodrama, is called the dramatization phase.

After a couple of stories are shared and played back, the therapist(s) should lead the group into the use of the long forms. When a shared story has several layers, relates to the group both as a whole and to its individuals, and has characteristics that sound strategic to the therapeutic team, it can be chosen for one of the long forms. In this case, after listening to the narrative, which can be interrupted by the therapist to ask the teller questions in order to get further details or feelings associated with the story, the rest of the group members (eventually divided into two subgroups, should the number of participants justify doing so) will have a few minutes to talk about how they will perform it.

Although improvisation will always be at the center of PPT, the playbackers can, especially in the first phases of the creation of the group, decide on issues such as who will play the protagonist role, what main scenes will be seen, and the main messages to be stressed. The therapist can help the group(s), not only during this preparation phase but even during the performance itself, through short, clear suggestions to the playbackers.

After the performance, the session will arrive at its closure, in which people will share their personal connections with the story or stories, the session as a whole, and even what they realized during the performance (or, in the case of the teller, while watching it). A closure ritual is frequently performed at the end of the session.

The present article is part of a project funded by the Portuguese Foundation for Science and Technology (FCT—2022.07713.PTDC) that aims to assess the impact of PPT in adults. For that, the team trained a group of 30 psychotherapists, from which 20 oriented groups of PPT. As part of the project, the research team published a handbook for the implementation of Psychotherapeutic Playback Theatre [73]. This document outlines the different stages and techniques that make up a PPT intervention. In doing so, it has helped the creation of PPT groups. Furthermore, this standardization and equivalence of procedures will allow researchers to evaluate the impact of both this and future interventions [73]. The groups were directed either by one, two, or three therapists, and 12 weekly sessions were delivered. In the following section, we will present the research design and procedures.

## 2. Materials and Methods

### 2.1. Design

The goal of PPT is two-fold: the promotion of well-being and the prevention and/or reduction of malaise, thus intervening in areas such as mental illness, adaptation to the varying challenges and conditions of life, promotion of a positive life purpose and hope, empowerment and personal development, and community development. In this study, we used a non-randomized pre- and post-test quasi-experimental design with an experimental group (EG) and a control group (CG) to assess the impact of this intervention. The independent variable considered in this study was the PPT intervention.

This psychotherapy requires professional training, and in the specific case of this project, a support handbook was used as a guide by all the therapists involved [25]. The control group (CG) was a comparison group composed of individuals who had subscribed to the open outreach session but, for various reasons (e.g., scheduling conflicts, familiarity with other members), did not join the PPT group. To make up 50 subjects for the CG, we included university students of similar age and gender to the EG. The treatment had an intervention duration of 2 to 3 weekly hours over 12 weeks. The intervention lasted 2 to 3 h per week over 12 weeks. The results of the follow-up are still being collected, with the process scheduled to be completed by the end of the current calendar year. For this reason, this follow-up data was neither presented nor used in the present analysis evaluating the impact of the PPT intervention.

### 2.2. Participants

The present study is based on the application of PPT to 9 psychotherapeutic groups in different regions of Portugal. These groups were all different in size, composition, and heterogeneity of the participants. This paper will, however, present an analysis of the participants as a whole and not the specificities of each group, with the 9 therapeutic groups being merged and thus forming the experimental group because our interest was to understand whether the effect was beneficial despite the heterogeneity of participants. Nevertheless, subjects were not randomly selected nor assigned to the research groups (CG—Control Group; EG—Experimental Group). Fifty (50) participants belong to the EG, and another set of 50 subjects belong to the CG. The average age for the CG and EG was 30.1 (SD = 14.7) and 37.1 (SD = 14.0), respectively. The CG comprised 39 females and 11 males, whereas the EG comprised 37 females, 12 males, and one non-binary person. The control group comprised 17 subjects with a mid/high-school level of education, 30 subjects with an undergraduate degree, and 3 with a postgraduate degree. As for the experimental group, the group comprised 14 subjects with a mid/high school level of education, 14 with an undergraduate degree, and 22 with a postgraduate degree. Additionally, both groups brought together subjects from the greater Lisbon area and the more northern part of Portugal, which included the cities of Leiria, Coimbra, Aveiro, and Figueira da Foz. Each psychotherapeutic group took place in different settings (e.g., hospitals, day centers, private clinics, university consultation centers) and was oriented by 1 to 3 group psychotherapists who received training in PPT. Finally, participants were assessed based on their overall distress and malaise and not pathology.

### 2.3. Measures

To validate the effectiveness of PPT as a clinical practice with empirical evidence, we used a protocol with instruments duly validated for the Portuguese adult population. The research protocol consists of evaluation scales to measure the effectiveness of the PPT intervention (PWBS and CORE-10) used in the pre-test, post-test, and monitoring of this research.

- **Psychological Well-being Scale** (PWBS, Ryff, 1989; Portuguese version, Novo, Duarte-Silva and Peralta, 1997 [74,75]) operationalizes the dimensions of positive psychological functioning related to personal growth and interpersonal involvement. These dimensions bring together essential characteristics for personality development and mental health, as identified by authors such as Jung, Rogers, Maslow, Allport, Neugarten, and Jahoda. The scale is made up of 6 nuclear scales/dimensions of normal development, in a positive and well-being sense, as follows: Self-Acceptance (assessing a positive attitude towards oneself), Positive Relationships with Others (connected to the presence of feelings of reciprocity, empathy, or intimacy with others), Mastery of the Environment (the feeling of being in control of life situations), Personal Growth (linked to the purpose of a continuous self-development and improvement), Objectives in Life (the presence of a sense of purpose and meaning in life), and Autonomy (connected to the ability of making decisions independently of other people’s opinions or social pressure). Taking into account the subscale to which each statement refers, they are presented alternately referring to favorable or unfavorable aspects of psychological well-being, measured on a 6-point Likert-type scale (from “I completely disagree” to “I completely agree”). Each subscale has half of the items stated in a positive way and the other half in a negative way to control response attitudes related to the tendency to acquiesce. The dimensions of the scale in its Portuguese version present Cronbach’s alpha coefficient values ranging between 0.71 and 0.82, values overlapping with those of the original scale.
- **CORE-10** (Clinical Outcome Routine Evaluation 10) (Mellor-Clark et al., 1999; Portuguese version, Sales et al., 2012 [76,77]) is a shortened version of the 34-item CORE-OM scale. Each item is evaluated on a scale that varies from “Never” to “Always or almost always”, with the extreme values ranging between 0 and 4 points, referring the participant to the experience of the previous week. It is an easy-to-use assessment that measures psychological distress, to be used for screening as well as throughout treatment to track progress. It contains items covering anxiety, depression, trauma, physical problems, functioning, and risk to self. Six of the items are from the problem domain, three from the functional domain, and one from the risk domain. The total score indicates a person’s level of discomfort and psychological distress. The internal consistency levels of the Portuguese CORE-OM scale are the same as those found in the total UK scale (α = 0.94); the internal consistency for CORE-10 in the original scale is α = 0.90.

### 2.4. Procedure

The current study was conducted in Portugal, and the Ethical Board of the university institute approved the ethical form submitted by the research team.

Participants responded to a paper and online invitation placed in psychotherapists’ clinics and care centers to participate in a PPT group. Therefore, they received no incentives to participate in the study, and they had different motivations and problems/difficulties. All participants were informed about the purpose of the study and asked to sign an informed consent form, which outlined their rights and assured them of anonymity and confidentiality in the handling of their data.

On the day of completing the questionnaires, all participants were once again given information about the purpose, objectives, and methods of the study, and data protection was ensured at all times. The questionnaires were distributed at two different times, i.e., before the intervention and after the intervention. In total, 200 questionnaires were distributed, 100 to the experimental group and 100 to the control group, 50 and 50, respectively, at each of the data collection moments. The questionnaires were self-administered and took around 15 min to complete.

As mentioned, this study is part of a larger project involving several studies. In this first study, we present data comparing the EG and CG, as well as pre- and post-test evaluations. 

The complete assessment process covered the following components:

- Pre- and post-test evaluation, meaning before and after the intervention (to detect changes or improvements after the intervention).
- Continuous evaluation of the process (this type of evaluation is fundamental for the development of psychotherapy). It assesses, for example, how the sessions are being experienced, and we used measures of the TPP process (HAT and Change Interview).

Follow-up data, continuous evaluation, and qualitative analysis will be presented in future articles. 

### 2.5. Statistical Analysis

For data analysis, the statistical program Jamovi 2.3.28 was used to conduct a descriptive and inferential analysis of the results of pre- and post-intervention (Psychotherapeutic Playback Theatre) scores that resulted from filling out the two questionnaires described in the measures section (PWBS and CORE 10). After screening, i.e., verifying that all participants answered every item from the questionnaires, the database was organized into a control group and an experimental group. Next, to test the reliability of the scales, Cronbach’s alpha test was used. The reliability values of the original scales were described in Section 2.3.

Using a statistical significance level of *p* < 0.05 (*) or *p* < 0.01 (**), each construct within each of the previously mentioned groups had its pre- and post-scores analyzed using either a paired samples *t*-test or a non-parametric Wilcoxon test, depending on whether the data met the assumption of normality. Upon analyzing the experimental group, the effect size was estimated using Cohen’s d, which was deemed small when the score fell between 0.2 and 0.49, medium when between 0.5 and 0.8, and large when greater than 0.8. Additionally, for the calculation of the effect size, Cohen’s d test was used [78].

## 3. Results

The control group was established to rule out the potential influence of other variables on the dependent variables assessed during the study. In doing so, we sought to lay the foundation for potentially establishing a relationship between the results found in the experimental group and their participation in the Psychotherapeutic Playback Theatre intervention. 

This being said, the results showed that the construct Well-being failed to meet the assumption of normality (*p* = 0.038) within the control group. As such, said data was further analyzed using the Wilcoxon non-parametric test (Table 1), which indicated no significant differences. A similar result was found for the construct CORE (via parametric testing), with no significant differences identified within these two constructs (Well-being and Psychological Distress/CORE). Consequently, such baselines will therefore strengthen the association between their corresponding scores within the experimental group and the latter’s participation in the PPT program. In addition, the results of the analysis indicated that the effect size is medium/large and large for the Well-Being and CORE scales, respectively, in the experimental group. As for the control group, the data analysis showcases a small effect size both for CORE and Well-being Scales. 

Concerning the six subscales that constitute the Psychological Well-being Scale (Table 1), the only constructs to showcase normality were Environment Mastery and Purpose in Life. Hence, in addition to the absence of normality in the global scale core of the PWBS, the data corresponding to the subscales Autonomy (*p* = 0.025), Personal Growth (*p* = 0.003), Positive Relations with others (*p* < 0.001), and Self-Acceptance (*p* = 0.011) also showcased an absence of normality. None of these constructs showcased significant differences between pre- and post-intervention scores in the control group.

In summary, no significant differences were found in the control group, either in the Psychological Well-Being construct and its six subscales or in the Psychological Distress score assessed through the CORE-10 scale. This, therefore, allows for a stronger analysis of the results from the experimental group, enabling the exploration of an association between the differences in pre- and post-intervention scores and the participants’ involvement in the PPT program.

**Figure 2:**
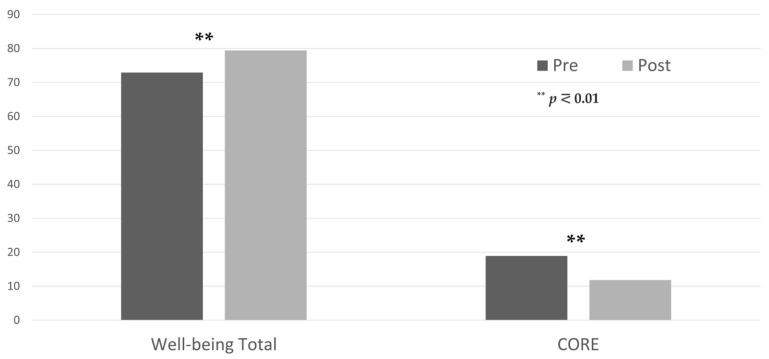
Differences between pre- and post-assessment of both the global scores of Psychological Well-being and CORE-10 scales of the experimental group.

**Figure 3:**
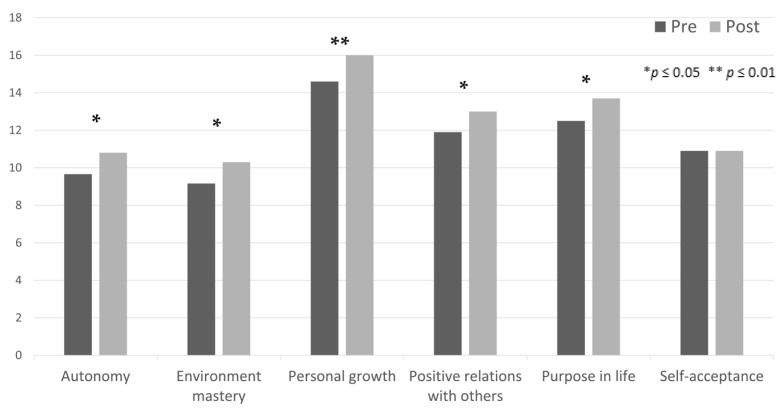
Differences between pre- and post-assessment of the six subscales of the Psychological Well-being Scales of the experimental group.

As for the experimental group, the results indicated normality in the constructs of Psychological Well-being (total score), Autonomy, Positive Relations with Others, Purpose in Life, and Self-Acceptance. The remaining constructs did not meet the assumption of normality (Environment Mastery—*p* = 0.002; Personal Growth—*p* < 0.001; and CORE—*p* = 0.036), thus requiring non-parametric testing. As presented in Table 1, all scores, except for the Self-Acceptance construct, showed significant differences when comparing pre- and post-intervention scores. Furthermore, the differences between pre- and post-program scores were highly significant for the CORE-10, the PWBS total score (see Table 1 and Figure 2), and the Personal Growth subscale (see Table 1 and Figure 3). Lastly, as shown by the mean scores (Table 1), all constructs displaying significant differences showed an increase in scoring, except for the CORE score, which showed a decrease in the assessed output.

## 4. Discussion

In this work, we present the results of an experimental group that received 12 sessions of Psychotherapeutic Playback Theatre and compare them to a control group. We assessed two main variables: Well-being (measured by PWBS, which is composed of a global scale and six sub-scales) and Psychological Distress (measured by CORE-10). We analyzed pre- and post-test values in both control and experimental groups.

Concerning the experimental group, upon comparing the values before and after the PPT intervention, we encountered statistically significant differences in Psychological Distress and the global Well-being Scale, as well as in its subscales (except for Self-Acceptance). No significant differences were found in the control group. These results suggest that the changes, both the enhancement of Well-being and diminishing of Psychological Distress, are associated with participation in the 12-session PPT program.

In conceptualizing the change mechanisms associated with PPT, we can identify the power of narratives, the group, action (using both standard action and improvised action), and relationships, and the positive forces associated with them [73]. We know that the sessions led by trained psychotherapists followed dynamics that facilitated these mechanisms. The implementation handbook [73], which was followed by the group leaders, not only suggested specific dynamics adapted to each of the group stages but also included exercises and warm-up routines that facilitated and put into action these mechanisms. The next step in understanding the specific dynamics of this particular psychotherapeutic process is to use the feedback given by both group participants and therapists. 

If we analyze in more detail the subscales of the Well-being construct that significantly changed after the treatment by relating them to what was proposed throughout the sessions, we can attempt to make connections between these aspects and the literature on this therapeutic approach, namely the works concerning change mechanisms in psychotherapy, be it individual or in group contexts. Throughout the 12 sessions, a group of strangers gradually developed deep connections with each other. Personal and, in most cases, deep and significant stories were shared, carefully listened to, and then transformed by the listeners into improvised theatrical forms with an important component of bodily and symbolic language. 

It is easy to connect this experience of deep sharing with others to a change in one’s perception of Personal Growth, noting that this was the dimension that reported the largest effect size in the experimental group, with a Cohen’s d of 0.861, considered high. Furthermore, it is also possible to connect this with our ability to be open to new experiences. The challenges they bring to the relationships maintained with oneself, others, and the world include enhancing the quality of our Positive Relations with others (seeing oneself as empathetic, affectionate, and intimate with others). Group cohesion, as conceived by Yalom and Leszc [79], is analogous to trust in the therapeutic relationship in individual psychotherapy. Lastly, with the latter being deemed one of the core mechanisms of therapeutic change, it seems to be clearly connected to the results outlined. 

Concerning Autonomy and Environmental Mastery, two of the Well-being subscales show very significant changes in the experimental group (but not in the control group), both can be easily connected to the atmosphere of improvisation. In such circumstances, although sometimes feeling challenged at the beginning, participants progressively learn to accept their perspectives on the stories being told. Their impulses to interpret the narratives (in the case of participants who playback the narratives) are accompanied by the feeling that sharing their life narratives (frequently about critical or vulnerable situations) is accepted, validated, or understood by others. As supported by their influence in psychodrama [64,65], the presence of a central place for improvisation and action in the core of PPT principles and practices provides us with a rationale to explain these positive changes in the participants.

Finally, concerning the dimension of Purpose in Life, which is closely associated with giving meaning to one’s life and life situations, it is easily connected to the atmosphere of helping each other in a significant manner. Other works with qualitative data from participants of PT sessions refer to increases in empathy and altruism [28,80,81].

Several of the aforementioned mechanisms involved in change can be connected to some of the common therapeutic factors of group psychotherapy [79], namely group cohesion, universality, interpersonal learning, and altruism, with the latter aligning with the enhancement of purpose in life among our participants. Furthermore, the empathetic atmosphere of active listening, facilitated throughout the sessions, strengthens the presence of Rogers’ necessary and sufficient conditions for therapeutic change [82].

Studying the impact of psychotherapy on clients’ quality of life, happiness, and well-being is fundamental. Many constructs and models are feasible. However, the psychological eudaimonic well-being model [74], generated some decades ago, addresses neglected aspects of positive functioning, such as purposeful engagement in life, realization of personal talents and capacities, and enlightened self-knowledge, while integrating several relevant constructs that are targeted by psychotherapy and personal development interventions, namely PPT. Increasing evidence supports the health-protective features of psychological well-being. In this realm, multiple studies employed measures of well-being to validate the effectiveness of diverse treatment interventions [83]. As expected, the majority of the psychological well-being scales showed improvement with PPT intervention. The exception is self-acceptance, which is a very important attitude underpinning mental health and, perhaps, one of the most difficult to change in relatively short interventions [84,85]. Self-acceptance implies changing beliefs about self-worth, such as acknowledging that you are a complex human being, including both making mistakes as well as attaining significant achievements, which is very difficult. Future research should analyze the impacts of this type of intervention in longer interventions that expand the number of sessions.

Psychotherapy started as a means to overcome mental illness and distress. Therefore, psychotherapy in this realm (and research that supports clinical interventions) should aim to decrease discomfort, disease, malaise, and distress. The CORE-10 is an acceptable, brief, and feasible instrument that has good psychometric properties and, therefore, is practical to use in clinical research with individuals experiencing common mental health problems and psychological distress. Data revealed a decrease in general psychological distress due to the PPT intervention, as shown in Table 1. This impact results from the PPT intervention, which utilizes the group setting, varied expressive resources, the sharing of significant narratives, and supports reframing. Nevertheless, future research should further analyze the impacts of this type of intervention in different specific pathologies.

## 5. Conclusions

We started this paper by stating that art can be therapeutic. When used following good empirically-based practices, it can form the base to psychotherapeutic approaches that enhance well-being and reduce suffering. Drama therapy and psychodrama have proven their therapeutic impact over the last few decades. To our knowledge, the effects of Psychotherapeutic Playback Theatre have previously only been reported in studies involving elderly citizens. With this work, we began to shed light on its impact on the quality of life in the adult population.

In general, participants in the PPT groups experienced improvements in their general Psychological Well-being and most of its subscales while also showing a reduction in psychological distress. Furthermore, we suggest that some of these changes may be associated with principles applied in PPT sessions, like building strong, significant relationships with others in closed, protected groups, promoting group cohesion, sharing personal narratives with the potential to give them new meaning, and the power of action, especially in the aesthetic and potentially symbolic space. In order to research what change mechanisms might be specific to PPT and which are common to other approaches, we need to combine quantitative and qualitative data. In future research, we will integrate this outcome data with process measures gathered throughout participation in the PPT groups.

## 6. Limitations and Suggestions for Future Research

Concerning some of the limitations of this study, and as stated before, to go deeper into understanding the mechanisms connected to these changes, we need to put together this data with the feedback from participants, which will be conducted in future work.

After relating the enhancement of Well-being and the reduction of Psychological Distress in the participants to the 12 sessions of PPT groups, researchers should start to connect these changes to the therapeutic mechanisms involved. To do this, connecting quantitative and qualitative data, as well as the use of new dependent variables and some mediating variables, would be beneficial. The use of groups receiving group treatments with different guidelines could help in understanding these mechanisms in a more specific way.

Furthermore, in order to construct strong models that could explain the changes, the number of participants should be higher. Until now, only adults have been studied, and it would be important to assess the impact of PPT on children and adolescents, as well as on other groups of participants, either from clinical or non-clinical populations. 

As stated before, it is not easy to guarantee the use of Randomized Controlled Trials, the golden standard of research, in the context of psychotherapy, as it brings ethical dilemmas in randomly assigning participants to control or experimental groups.

The CORE-10 is ideally suited to quick client assessments in a wide range of primary care settings. As such, it has a specific purpose that differs from the CORE-OM, which, however, should remain the preferred measure for detailed evaluation at intake (i.e., assessment) and at moderately spaced intervals throughout the course of medium- and longer-term psychological therapies.

Finally, the exclusive use of self-report assessment instruments has well-known limitations. Bringing in other forms of assessment would further enhance the validity of these studies.

To conclude, a paper based on a scientific study on the impacts of expressive arts by addressing its limitations and inspirations for the future is, at the same time, an editorial academic requirement and a good opportunity to express some of the personal gains we have experienced, both as scientists, artistic researchers, and therapists, along this path. Life is a journey marked by limits that simultaneously serve as scaffolds to our development and flourishing or prisons to our potential. Art does not save us from pain, injustice, or war. But using the arts to express our grief, suffering, desires, dreams, and imagination, and to use our expressive potential to share our narratives and/or transform them into different artistic formats, might make our common world more bearable, beautiful, empathic, and meaningful.

## Figures and Tables

**Table 1:** Results of the parametric (*t*-test) and non-parametric (Wilcoxon) testing and effect size of CORE-10 and the Well-being total scale and subscales within the control group and the experimental group.

		Control Group				Experimental Group			
		M	SD	*p*	Effect Size	M	SD	*p*	Effect Size
CORE ^b^	pre-test	15.2	7.63	0.114	0.23	18.9	5.98	<0.001 **	0.86
	post-test	13.9	8.14			11.8	6.25		
Well-being Total ^a^	pre-test	77.0	11.9	0.180	−0.23	72.9	12.5	<0.001 **	−0.69
	post-test	78.1	12.1			79.4	9.32		
Autonomy ^a^	pre-test	11.7	3.38	0.177	−0.23	9.66	3.44	0.004 *	−0.43
	post-test	12.0	3.17			10.8	2.81		
Environment mastery ^b^	pre-test	10.1	3.09	0.219	−0.18	9.16	3.37	0.010 *	−0.45
	post-test	10.5	3.07			10.3	2.94		
Personal growth ^a,b^	pre-test	15.1	2.49	0.834	0.04	14.6	2.43	<0.001 **	−0.89
	post-test	15.2	2.04			16.0	1.62		
Positive relations with others ^a^	pre-test	12.5	3.58	0.965	0.01	11.9	3.53	0.016 *	−0.35
	post-test	12.5	3.65			13.0	2.80		
Purpose in life	pre-test	13.7	2.90	0.550	−0.09	12.5	3.16	0.002 *	−0.46
	post-test	13.8	2.88			13.7	2.86		
Self-acceptance ^a^	pre-test	13.2	2.60	0.982	0.01	10.9	1.45	0.933	0.01
	post-test	13.2	2.82			10.9	1.58		

^a^ Wilcoxon (non-parametric) testing for the control group; ^b^ Wilcoxon (non-parametric) testing for the experimental group; * *p* ≤ 0.05; ** *p* ≤ 0.01.

## Data Availability

Data are contained within the article.
